# Comparison of Selected Therapies Within the Step-Up Therapeutic Approach in Canine Chronic Inflammatory Enteropathy

**DOI:** 10.3390/ani16203215

**Published:** 2026-10-13

**Authors:** Lukas Kunay, Mária Fialkovičová, Marta Benková, Csilla Tóthová, Nicol Kořená, Simona Gomulcová, Martina Vargová, Alena Micháľová, Branislav Lukáč, Bohumil Bielik, Radka Staroňová

**Affiliations:** 1 Small Animals Clinic, University of Veterinary Medicine and Pharmacy in Košice, Komenského 73, 041 81 Košice, Slovakia; 2 Faculty of Mining, Ecology, Process Control and Geotechnologies, Technical University, Letná 1/9, 042 00 Košice, Slovakia; 3 Clinic of Ruminants, University of Veterinary Medicine and Pharmacy in Košice, Komenského 73, 041 81 Košice, Slovakia

**Keywords:** CIE, CCECAI, chronic enteropathy, therapy, dog, budesonide, probiotics, faecal calprotectin

## Abstract

Canine chronic inflammatory enteropathy (CIE) frequently requires a sequential therapeutic approach in which treatment is escalated according to clinical response. This prospective, open-label clinical study evaluates selected dietary, antimicrobial, microbiome-directed, and corticosteroid interventions used within a step-up treatment strategy in dogs with CIE under routine clinical conditions. Fifty-six dogs were enrolled. Clinical response was assessed using the Canine Chronic Enteropathy Clinical Activity Index (CCECAI), together with faecal calprotectin, serum folate, and serum cobalamin concentrations. In the dietary stage, both novel-protein and hydrolysed diets significantly reduced CCECAI and faecal calprotectin. Clinical remission, defined as a final CCECAI score ≤ 3, was achieved in 64% and 60% of dogs, respectively. Baseline-adjusted analyses likewise showed no significant between-group differences in CCECAI, faecal calprotectin, or cobalamin. In dogs receiving second-line treatment, both metronidazole and probiotics combined with fibre significantly reduced CCECAI and faecal calprotectin, while baseline-adjusted comparisons demonstrated no significant differences between treatments for the evaluated outcome. In dogs receiving corticosteroid-containing treatment, substantial numerical reductions in CCECAI and faecal calprotectin were observed in both the prednisone-containing and budesonide-containing regimens; however, the very small subgroup sizes and concomitant dietary, probiotic, and fibre interventions precluded definitive conclusions regarding the specific effects or comparative efficacy of the individual corticosteroids. These findings provide real-world clinical evidence supporting a structured step-up approach to canine CIE and provide a rationale for larger, adequately powered controlled studies to determine whether non-antibiotic microbiome-directed strategies and locally acting corticosteroid-containing therapy can reduce reliance on empirical antimicrobial treatment and systemic corticosteroids, thereby minimising the potential adverse consequences associated with these therapies.

## Simple Summary

Chronic inflammatory enteropathy (CIE) is a common gastrointestinal disorder in dogs and often requires treatment to be adjusted step by step according to the individual patient’s response. This study evaluates selected dietary, microbiome-directed, antimicrobial, and corticosteroid treatments used in clinical practice. Both novel-protein and hydrolysed diets markedly improved clinical signs and reduced faecal calprotectin. In dogs requiring further treatment, both metronidazole and probiotics combined with fibre produced substantial clinical improvements. This finding is particularly relevant because effective non-antibiotic strategies may help reduce unnecessary antimicrobial use. In dogs with more severe disease requiring corticosteroids, both prednisone-containing and budesonide-containing treatments were associated with marked numerical improvement, although the small number of dogs did not allow definitive comparison of their efficacy. An important feature of this study is that it reflects real clinical practice, in which treatments are introduced sequentially and adapted according to patient response.

## 1. Introduction

Chronic nonspecific enteropathy in dogs is a common cause of persistent or recurrent gastrointestinal problems in small-animal clinical practice. It typically manifests as chronic diarrhoea, vomiting, changes in appetite, borborygmi, excessive flatulence, and progressive weight loss. The severity of the clinical signs may range from mild, intermittent episodes to conditions that markedly affect the patient’s overall body condition [1,2,3,4,5].

The aetiology of this disease is currently considered multifactorial in veterinary medicine. The major factors playing a key role in its onset and progression include the host’s genetic predisposition, the composition of the intestinal microbiota (dysbiosis), diet, and environmental factors, which collectively lead to dysregulation of the intestinal immune system [6,7,8,9].

As this is a diagnosis by exclusion, the primary objective of the diagnostic approach is the systematic exclusion of other known causes of chronic gastrointestinal symptoms, including infectious, parasitic, neoplastic, endocrine, and metabolic diseases [2,10,11,12,13].

Scoring systems such as the CIBDAI (Canine Inflammatory Bowel Disease Activity Index) and CCECAI (Canine Chronic Enteropathy Clinical Activity Index) are used in both clinical practice and research to objectively assess the severity of clinical signs. These systems evaluate parameters such as activity, appetite, vomiting, stool consistency, defecation frequency, and weight loss, while the CCECAI also includes serum albumin concentration and the presence of ascites or oedema [6,14,15,16].

Serum cobalamin (vitamin B12) and folate (vitamin B9) concentrations are commonly used as non-invasive functional markers in dogs with chronic gastrointestinal disease. Reduced concentrations may occur in dogs with chronic inflammatory enteropathy and can reflect disturbances in intestinal absorption. In particular, hypocobalaminemia is clinically relevant because it has been associated with more severe disease and poorer outcomes and may indicate a need for cobalamin supplementation. In contrast, serum folate is a less specific marker, as its concentration may also be influenced by dietary intake, intestinal dysbiosis, and cobalamin status. Therefore, these markers can help assess disease severity and provide useful baseline information about the extent of enteropathy, even when their values remain within standard reference intervals [2,17,18,19,20,21,22,23,24].

Faecal calprotectin is a non-invasive biomarker of intestinal inflammation that has been associated with disease severity and may provide additional information when monitoring treatment response in dogs with chronic inflammatory enteropathy [2,25,26,27].

In current veterinary medicine, chronic inflammatory enteropathy is classified retrospectively based on the response to therapeutic interventions. Food-responsive enteropathy (FRE), or diet-responsive enteropathy, is defined as a condition in which clinical signs completely resolve or improve significantly following the introduction of an elimination diet [2,10,28,29].

An elimination diet is considered the cornerstone of the diagnosis and treatment of chronic enteropathy. Given the high prevalence of FRE, an elimination diet should be the first therapeutic step for most clinically stable dogs with chronic gastrointestinal signs [2,10,30]. Hydrolysed and novel-protein diets are the most commonly used dietary strategies. Hydrolysed diets contain proteins broken down into small peptides and are often preferred when the dietary history is unclear because of their high digestibility and reduced risk of previous sensitisation [2,31,32].

Microbiome-modulation responsive enteropathy (MmRE), previously often referred to as antibiotic-responsive enteropathy (ARE), is a form of canine chronic enteropathy [2,10,31]. Metronidazole and tylosin have traditionally been used in dogs with an insufficient response to dietary therapy, although their effects may reflect antimicrobial, immunomodulatory, and microbiome-modifying properties [33]. However, routine empirical antibiotic use is increasingly questioned because clinical relapse is common after treatment withdrawal, while repeated or prolonged antimicrobial administration can cause persistent disruption of the intestinal microbiome and contribute to antimicrobial resistance [10]. The MmRE category includes dogs that respond clinically to therapeutic interventions aimed at restoring eubiosis and microbiome function without the use of antibiotics. These interventions include the administration of prebiotics, probiotics, and synbiotics, as well as faecal microbiota transplantation (FMT) [10,34].

Steroid-responsive enteropathy (SRE) represents a specific subgroup of chronic inflammatory enteropathies (CIEs) in dogs [1,2]. Unlike food-responsive enteropathy, immune dysregulation in SRE is sufficiently severe to require pharmacological immunosuppression [2,10].

Budesonide currently represents an important alternative to conventional glucocorticoids in veterinary gastroenterology for the treatment of chronic inflammatory bowel disease or, more specifically, immunosuppressant-responsive enteropathy. A key characteristic of budesonide is its extensive first-pass hepatic metabolism, during which approximately 90% of the absorbed drug is biotransformed into metabolites with minimal biological activity, specifically 16-alpha-hydroxyprednisolone and 6-beta-hydroxybudesonide. This pharmacokinetic property allows a potent local anti-inflammatory effect in the intestines while reducing systemic complications [35,36,37].

The traditional reliance on histopathology of intestinal biopsies as the first step of diagnosis is increasingly being challenged. Recent research highlights a weak correlation between histopathology scores and clinical disease activity. In addition, there is significant interobserver variability among pathologists in the assessment of specimens. As a result, the field is moving towards precision medicine, with biomarkers, molecular diagnostics, and advanced imaging modalities being brought to the fore to gain a more precise functional and prognostic understanding of each patient [38,39,40].

Non-responsive enteropathy (NRE), also referred to as refractory enteropathy, is a severe subgroup of chronic enteropathies in dogs. It is defined by a failure to achieve a clinical response to sequential therapeutic trials involving elimination diets, antimicrobial treatment or microbiome modulation, and standard immunosuppressive therapy [2,10,41].

In the present study, we aimed to evaluate, under routine clinical conditions, the clinical and biomarker responses associated with selected alternative treatments at successive stages of a step-up therapeutic approach.

## 2. Materials and Methods

### 2.1. Case Selection

Dogs (*n* = 56) examined between September 2021 and December 2025 at the Small Animal Clinic, Department of Internal Medicine, University of Veterinary Medicine and Pharmacy in Košice, were included in this study.

The dogs presented with signs of chronic enteropathy, such as chronic diarrhoea lasting for at least three weeks, weight loss, vomiting, abdominal pain, and similar clinical signs. The owners signed informed consent forms approving the inclusion of their dogs in the study.

After a detailed medical history was obtained, each enrolled patient underwent a thorough examination to exclude all other potential causes of chronic diarrhoea, including infectious diseases, extra-gastrointestinal disorders, and other conditions.

A complete health assessment was performed for all patients, including a complete blood count using an IDEXX ProCyte DX™ analyser (IDEXX Laboratories, Inc., Westbrook, ME, USA) and serum biochemistry using a COBAS C 111 analyser (Roche Diagnostics International Ltd., Rotkreuz, Switzerland). The diagnostic evaluation included measurement of serum cobalamin, measurement of folate concentrations, basal cortisol measurement, parasitological examination of faeces, urinalysis, assessment of nutritional status using the Body Condition Score, and a complete ultrasonographic examination focused primarily on the gastrointestinal tract.

In selected patients with very severe conditions, endoscopic examination was performed, including the collection of biopsy samples for histopathological evaluation. Dogs diagnosed with another underlying disease were excluded from the study.

Faecal calprotectin concentrations were determined using the commercially available Canine Calprotectin ELISA Kit (MyBioSource, Inc., San Diego, CA, USA). The analysis was performed using a quantitative sandwich immunoassay method. In healthy dogs, the reference range is 10.6–20.9 ng/mL.

Serum folate and cobalamin measurements were performed using a chemiluminescence assay (Access Immunoassay System; Beckman Coulter, Inc., Brea, CA, USA). Reference range for folate is 6.5–18.6 ng/mL. Reference range for cobalamin is 400–1000 pg/mL.

The study population consisted of 56 dogs of various breeds, aged 1–16 years (mean age: 6.0 ± 3.7 years). Twenty-nine dogs were female, including 8 spayed females, and 27 were male, including 1 neutered male. The breed distribution was as follows: Yorkshire Terrier (*n* = 8), German Shepherd Dog (*n* = 7), mixed-breed dogs (*n* = 7), Golden Retriever (*n* = 5), Staffordshire Bull Terrier (*n* = 4), Maltese (*n* = 3), hairless dogs (*n* = 2), Poodle (*n* = 1), French Bulldog (*n* = 1), Rhodesian Ridgeback (*n* = 1), Labrador Retriever (*n* = 1), Schnauzer (*n* = 1), Chihuahua (*n* = 1), Welsh Terrier (*n* = 1), Puli (*n* = 1), Cavalier King Charles Spaniel (*n* = 1), Weimaraner (*n* = 1), Bedlington Terrier (*n* = 1), Shih Tzu (*n* = 1), Irish Setter (*n* = 1), Tatra Hound (*n* = 1), Whippet (*n* = 1), Belgian Shepherd Dog (*n* = 1), Cane Corso (*n* = 1), Dachshund (*n* = 1), Lhasa Apso (*n* = 1), and Bull Terrier (*n* = 1).

### 2.2. Methods

Only patients with chronic inflammatory enteropathy were included in the study; others were excluded from the study. Each patient underwent baseline CCECAI scoring during the initial examination at time point T0. The patients were subsequently divided into four categories according to disease severity based on their CCECAI scores. Several clinical parameters were evaluated, with each parameter assigned a score ranging from 0 to 3. A higher score indicated more severe clinical manifestations of the disease. The individual scores were then summed, and the total CCECAI score was evaluated and interpreted.

Of the total number of patients studied (*n* = 56), fifty of them had a CCECAI score of 4 to 11. They showed mild and moderate-to-severe enteropathy. Six patients with severe-to-very severe enteropathy had a CCECAI score of more than 12.

In the study group of patients with CCECAI scores from 4 to 11 (*n* = 50) with mild, moderate, and severe enteropathy, the effects of changing the therapeutic diet were evaluated in the first phase of treatment protocol. A hydrolysed elimination diet and a novel-protein diet were used. For both therapeutic diets, eligible dogs were allocated alternately to one of the two treatment diet options, independently of individual patient characteristics or disease severity.

The hydrolysed diet used was Brit Grain-Free Veterinary Diet Dog Hypoallergenic Hydrolyzed (Brit Veterinary Diet), formulated with peas (30%), pea protein (20%), hydrolysed poultry protein (14%, molecular weight < 5000 Da). Its declared analytical composition included crude protein (22.0%), crude fat (15.0%), crude fibre (3.2%), and crude ash (8.0%).

The novel-protein diet was Hill’s Science Plan Hypoallergenic Adult Dog Food with Salmon (Hill’s Pet Nutrition). The principal ingredients included dried potatoes (21%), potato starch, dried peas, salmon meal (9%), and pea protein. The declared analytical composition included protein (19.9%), fat (14.2%), crude fibre (2.1%), and crude ash (4.8%). Salmon was the principal intact animal protein source.

After four weeks, at time point T1, a follow-up examination was performed, and the CCECAI score was reassessed.

Patients whose CCECAI scores improved were classified as having food-responsive enteropathy and were assigned to the FRE group (*n* = 29). Patients whose clinical condition, as assessed via the CCECAI score, did not improve were subsequently administered a second diet containing hydrolysed protein source. Farmina Vet Life UltraHypo (Farmina Pet Foods) was used. This diet contains rice starch as the carbohydrate source and hydrolysed fish protein as the protein source. Its declared analytical composition includes crude protein (18.0%), crude fat (15.0%), crude fibre (1.2%), and crude ash (5.3%).

After a further four weeks, at time point T2, another follow-up examination was performed. Dogs that responded positively to the second dietary change (*n* = 4) were also assigned to the FRE group. Overall, the FRE group consisted of 33 patients.

Dogs whose CCECAI scores did not improve continued with the next therapeutic protocol. This protocol consisted of the administration of metronidazole (Entizol tablets) at a dose of 10 mg/kg twice daily for 10 days or probiotics (Purina Pro Plan FortiFlora for dogs) combined with fibre (Psyllium), which was administered once daily at a dose of half a tablespoon for small dogs and one tablespoon for medium-sized and large dogs in the long term. This treatment was administered to the remaining patients (*n* = 17). In both therapeutic steps, eligible dogs were allocated alternately to one of the two treatment options, independently of individual patient characteristics or disease severity.

After four weeks, at time point T3, a follow-up examination was performed, and the CCECAI score was reassessed. The response to treatment was defined in the same manner as in the previous therapeutic protocol. Dogs that responded positively to antibiotics or to probiotics combined with fibre (*n* = 14) were assigned to the microbiota-modulation-responsive enteropathy group (MmRE).

Because some patients (*n* = 3) from the dog group did not respond to previous therapeutic procedures, a further therapeutic step was followed by corticosteroid treatment.

These patients (*n* = 3) had worsening CCECAI scores and were finally followed up together with a group of six dogs with very severe enteropathy, for which the CCECAI score was already higher than 12 at the initial health assessment.

In a group of dogs with very severe clinical conditions and very severe enteropathy with CCECAI score above 12 (*n* = 6), a combination of ultrahypoallergenic dry diet (Farmina Vet Life UltraHypo Canine), probiotics (Purina Pro Plan FortFlora for dogs) and fibre (psyllium) was used.

Treatment for all patients in this group (*n* = 6 + 3) consisted of either budesonide (Entocort capsules) or prednisone. Budesonide was administered at 3 mg/m^2^ to dogs for one month. The dose was subsequently reduced to one-half of the initial dose for two weeks and then to one-quarter of the initial dose for a further two weeks. Prednisone was administered at 2 mg/kg twice a day for one month; this was then tapered to 1 mg/kg for two weeks and 0.5 mg/kg for a further two weeks.

In both therapeutic steps, eligible dogs were allocated alternately to one of the two treatment options, independently of individual patient characteristics or disease severity.

These patients also underwent endoscopic examination involving the collection of biopsy samples for histopathological evaluation to exclude lymphoma. In all cases, the intestinal biopsies were consistent with lymphoplasmacytic enteritis, and no histological evidence of intestinal lymphoma was identified. The response to treatment was defined in the same manner as in the previous therapeutic protocols. Dogs that responded positively to corticosteroid-containing treatment, either administered alone or in combination with an ultra-hypoallergenic dry diet, probiotics, and fibre (*n* = 8), were assigned to the steroid-responsive-enteropathy group (SRE). No cobalamin supplementation was administered during the study.

The dog whose CCECAI score did not improve (*n* = 1) had non-responsive enteropathy (NRE).

**Figure 1:**
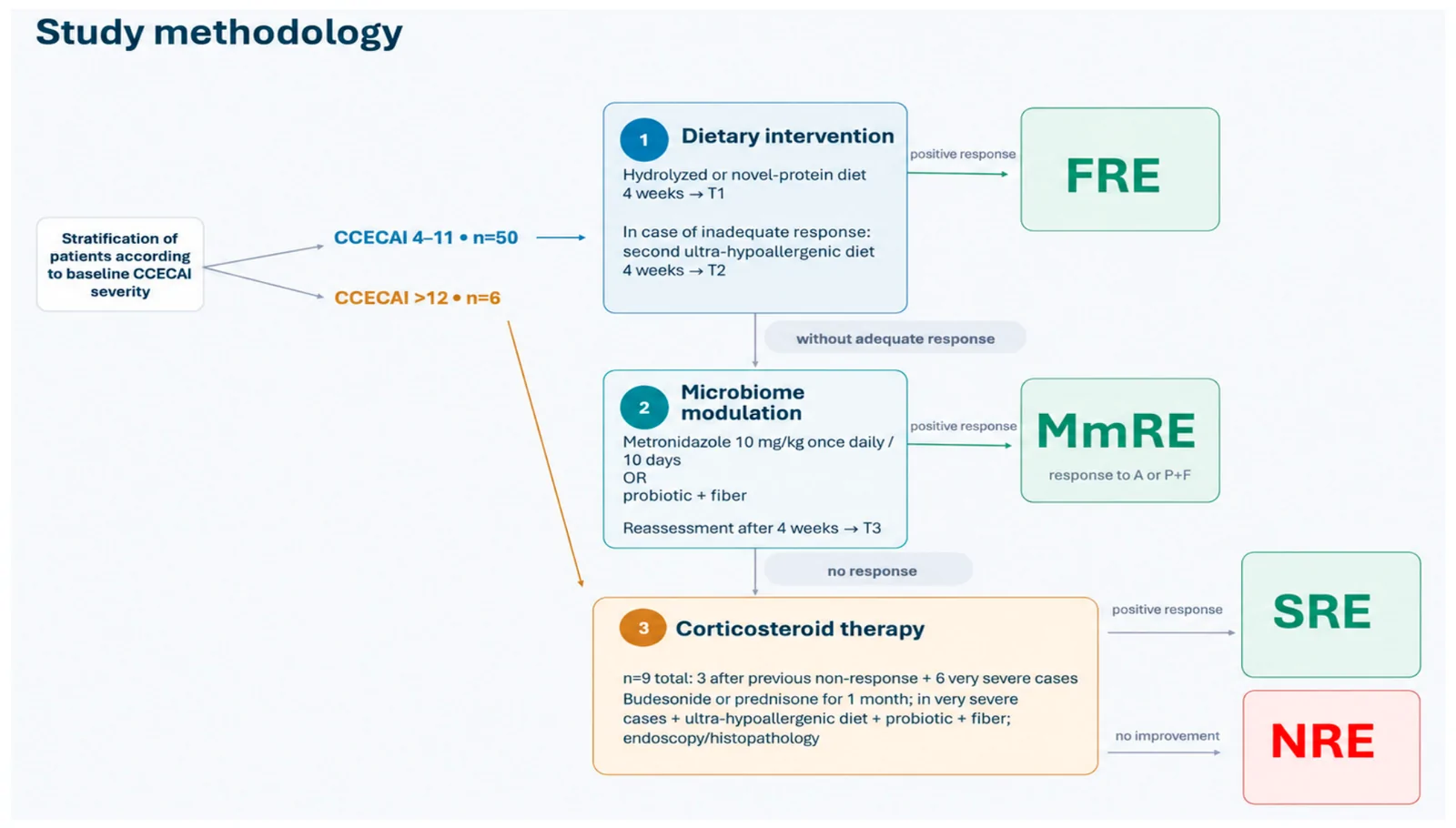
Flow diagram of the step-up therapeutic protocol. Flow diagram illustrating patient progression through the step-up therapeutic protocol according to baseline CCECAI severity and subsequent treatment response. Dogs with mild to moderate disease activity underwent dietary intervention followed, when necessary, by microbiome-modulating therapy and corticosteroid-containing treatment, whereas dogs with very severe disease activity proceeded directly to corticosteroid-containing therapy. According to treatment response, dogs were classified as having food-responsive enteropathy (FRE), microbiome-modulation-responsive enteropathy (MmRE), steroid-responsive enteropathy (SRE), or non-responsive enteropathy (NRE).

A flow diagram summarising patient progression through the step-up therapeutic protocol and the resulting treatment-response classifications is provided in Figure 1.

### 2.3. Statistical Methods

Statistical evaluation was performed using Minitab ver. 15 and Microsoft Excel 365. Continuous variables were initially assessed for normality using the Shapiro–Wilk test, and homogeneity of variance was evaluated using Levene’s test. Data are presented as means ± standard deviations (SDs) for continuous variables and as absolute numbers and percentages for categorical variables. All statistical tests were two-sided, and statistical significance was defined as *p* < 0.05. For baseline comparisons between treatment groups, the independent-samples *t*-test was used for continuous variables meeting the assumptions of normality and homogeneity of variance. The non-parametric Mann–Whitney U test was used when the assumption of normality was not satisfied. Categorical variables, including sex distribution, were compared using Fisher’s exact test. To evaluate changes within individual treatment groups between baseline and post-treatment assessments, paired analyses were performed. Laboratory variables with normally distributed paired differences were analysed using the paired-samples *t*-test. Changes in CCECAI, which was treated as an ordinal clinical score, as well as laboratory variables with non-normally distributed paired differences, were analysed using the Wilcoxon signed-rank test. For direct comparisons of treatment effects between groups, baseline-adjusted regression models were used to account for differences in baseline measurements. For CCECAI, between-group differences in post-treatment scores were evaluated using baseline-adjusted ordinal logistic regression, with treatment group as the explanatory variable and baseline CCECAI as a covariate. For continuous laboratory biomarkers (faecal calprotectin, serum folate, and serum cobalamin), baseline-adjusted linear regression models were fitted using the corresponding post-treatment value as the dependent variable, treatment group as the explanatory variable, and the baseline value as a covariate. Robust standard errors were used to reduce sensitivity to heteroscedasticity. Treatment effects are reported with 95% confidence intervals. The proportions of dogs achieving clinical remission, defined as a final CCECAI score ≤ 3, were compared using Fisher’s exact test. Because of the very small sample sizes in the corticosteroid subgroups (*n* = 4 per group for paired analyses), standard asymptotic assumptions were considered unreliable. Consequently, within-group changes in these subgroups were evaluated using the exact Wilcoxon signed-rank test, and direct between-group comparisons of individual changes were performed using the exact Mann–Whitney U test. Baseline-adjusted regression analyses in the corticosteroid groups were considered exploratory because of the limited sample size. To account for multiple testing across related outcomes, *p* values from the between-treatment comparisons were adjusted using the Holm method. Both unadjusted and Holm-adjusted *p* values were considered when interpreting the results, with primary conclusions based on the adjusted values.

## 3. Results

### 3.1. Analysis of Changes in CCECAI Scores and Comparison of Selected Parameters Following the Use of a Hydrolysed Diet and a Novel-Protein Diet

A total of 50 patients were included in the evaluation of the effects of a hydrolysed diet and a novel-protein diet. No statistically significant differences in baseline age, sex distribution, CCECAI score, faecal calprotectin, serum folate, or serum cobalamin concentrations were observed between the novel-protein and hydrolysed diet groups (all *p* > 0.05). The baseline characteristics of the treatment groups are given in Table 1.

In dogs fed the novel-protein diet, the mean CCECAI score decreased from 5.96 ± 1.90 at baseline to 2.56 ± 2.90 after treatment. A significant within-group reduction in CCECAI was observed (*p* = 1.45 × 10^−6^). A final CCECAI score of ≤3 was achieved for 16 of 25 dogs (64%). Faecal calprotectin concentrations also decreased significantly, falling from 32.52 ± 11.39 ng/mL to 17.39 ± 11.86 ng/mL (*p* = 0.000336). Serum folate concentrations increased from 11.20 ± 4.06 ng/mL to 12.46 ± 4.53 ng/mL, whereas serum cobalamin concentrations decreased from 483.36 ± 255.01 pg/mL to 438.24 ± 143.54 pg/mL; neither change was statistically significant.

In dogs fed the hydrolysed diet, the mean CCECAI score decreased from 6.32 ± 1.57 at baseline to 3.08 ± 3.26 after treatment (*p* = 1.85 × 10^−5^). A final CCECAI score of ≤3 was achieved for 15 of 25 dogs (60%). Faecal calprotectin concentrations decreased significantly from 35.40 ± 14.13 ng/mL to 17.08 ± 10.97 ng/mL (*p* = 8.15 × 10^−6^). Serum folate concentrations decreased from 12.19 ± 6.52 ng/mL to 9.39 ± 3.62 ng/mL, while serum cobalamin concentrations increased from 400.28 ± 199.96 pg/mL to 445.60 ± 145.33 pg/mL. These within-group changes were not statistically significant.

**Figure 2:**
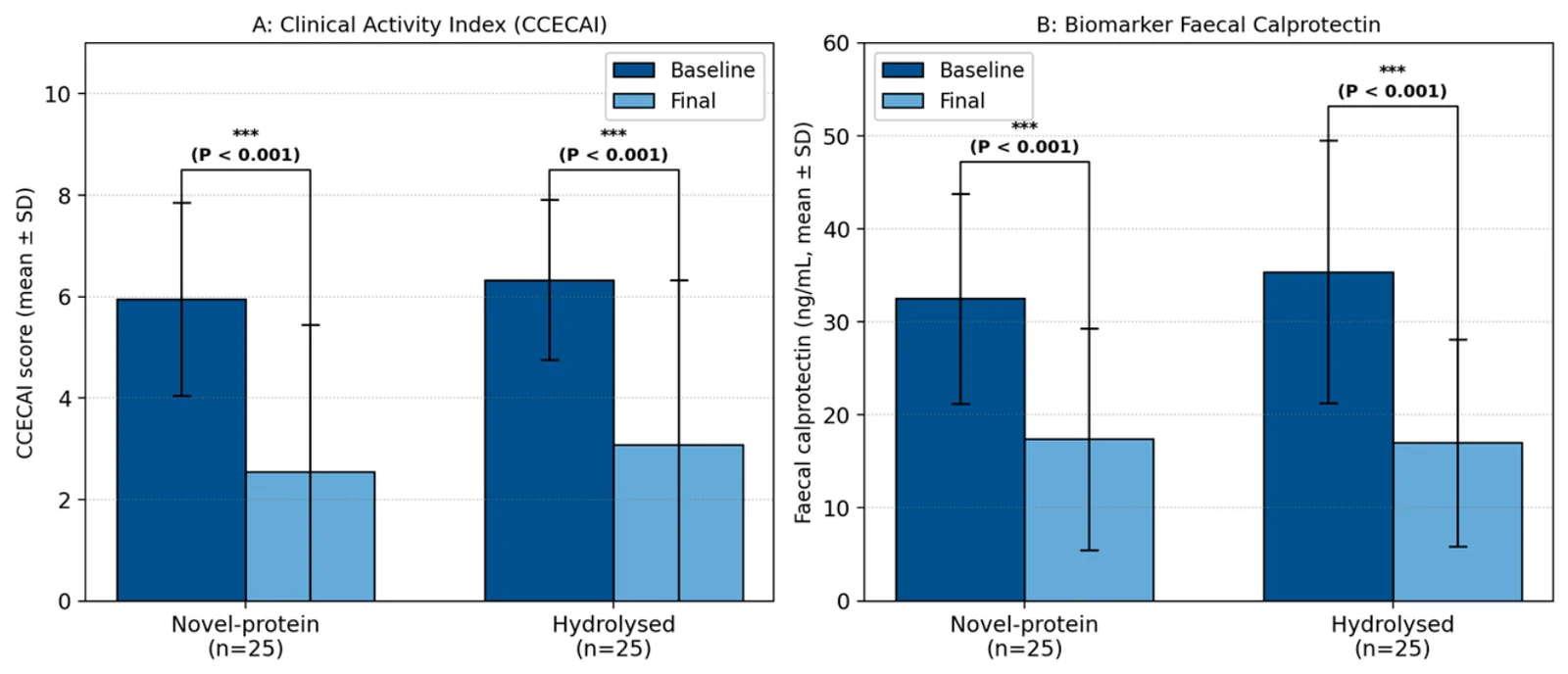
Clinical and biomarker responses to dietary interventions. Comparison of baseline (dark blue) and post-treatment (light blue) values in dogs fed a novel-protein diet (*n* = 25) or a hydrolysed diet (*n* = 25). (**A**) Canine Chronic Enteropathy Clinical Activity Index (CCECAI) scores. (**B**) Faecal calprotectin concentrations (ng/mL). Data are presented as means ± standard deviations (SDs). Asterisks (***) indicate highly significant within-group reductions from baseline (*p* < 0.001) evaluated by the Wilcoxon signed-rank test for CCECAI and paired-samples *t*-tests for faecal calprotectin.

A comparison of baseline and post-treatment values of CCECAI and calprotectin in dogs fed a novel-protein diet or a hydrolysed diet is given in Figure 2.

The proportion of dogs that achieved a final CCECAI score of ≤3 did not differ significantly between the novel-protein and hydrolysed diet groups (64% vs. 60%, respectively; Fisher’s exact test, *p* = 1.00).

Baseline-adjusted comparisons between the two dietary groups showed no statistically significant differences in post-treatment CCECAI, faecal calprotectin, or serum cobalamin concentrations. For CCECAI, the baseline-adjusted ordinal regression model showed no significant association between dietary treatment and the final CCECAI score (OR = 0.74; 95% CI, 0.27–2.05; *p* = 0.562). The adjusted between-group difference in faecal calprotectin was 0.30 ng/mL (95% CI, −6.36 to 6.97; *p* = 0.929), and the adjusted difference in serum cobalamin was 6.01 pg/mL (95% CI, −80.00 to 92.03; *p* = 0.891). In contrast, serum folate concentrations were higher in the novel-protein group after adjustment for baseline values, with an adjusted between-group difference of 3.09 ng/mL (95% CI, 0.62–5.56; unadjusted *p* = 0.014); however, this difference did not remain statistically significant after correction for multiple comparisons (Holm-adjusted *p* = 0.056). Detailed baseline and post-treatment values, within-group changes, effect estimates, and 95% confidence intervals are presented in Table 2.

### 3.2. Analysis of Changes in CCECAI Scores and Comparison of Selected Parameters Following the Use of Antibiotics or Probiotics Combined with Fibre

In this part of the study, we evaluated the effects of two different treatment protocols—the administration of metronidazole (*n* = 8) and a combination of probiotics and fibre (*n* = 9)—on changes in the CCECAI clinical index in the monitored patients.

No statistically significant differences in baseline age, sex distribution, CCECAI score, faecal calprotectin, serum folate, or serum cobalamin concentrations were observed between the metronidazole and probiotics + fibre groups (all *p* > 0.05). The baseline characteristics of the treatment groups are given in Table 3.

In dogs given metronidazole, the mean CCECAI score decreased from 6.38 ± 1.30 at baseline to 2.63 ± 3.54 after treatment, representing a significant within-group reduction (*p* = 0.031). A final CCECAI score of ≤3 was achieved in 7 of 8 dogs (87.5%). Faecal calprotectin concentrations decreased significantly from 40.86 ± 7.82 ng/mL to 10.10 ± 11.04 ng/mL (*p* = 0.000246). Serum folate concentrations increased from 7.41 ± 2.53 ng/mL to 11.45 ± 2.99 ng/mL (*p* = 0.011), whereas serum cobalamin concentrations changed from 457.75 ± 281.48 pg/mL to 424.88 ± 109.51 pg/mL, without a statistically significant difference.

**Figure 3:**
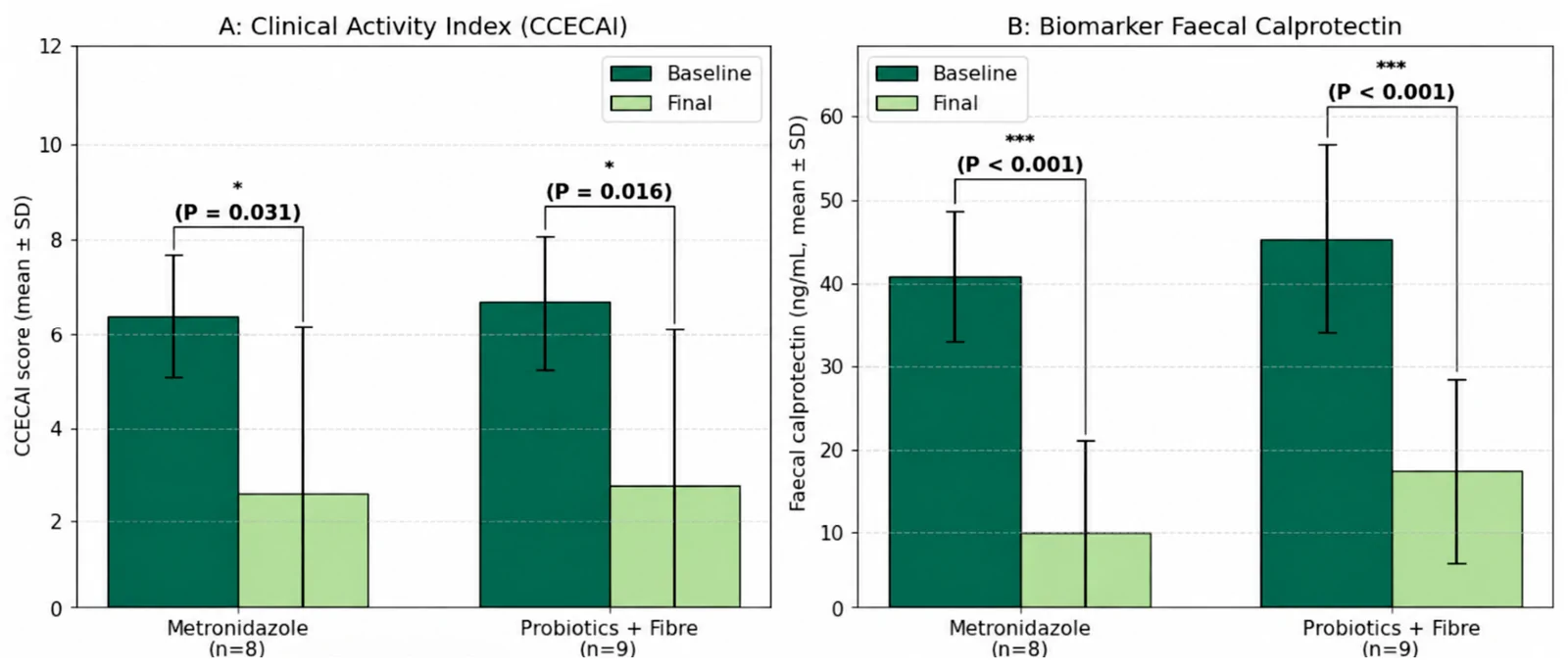
Clinical and biomarker responses to the metronidazole and probiotics-with-fibre treatments. Comparison of baseline (dark green) and post-treatment (light green) values in dogs treated with metronidazole (*n* = 8) or a combination of probiotics and fibre (*n =* 9). (**A**) Canine Chronic Enteropathy Clinical Activity Index (CCECAI) scores. (**B**) Faecal calprotectin concentrations (ng/mL). Data are presented as means ± standard deviations (SDs). Significant within-group improvements are marked with asterisks: * indicates *p* < 0.05 and (***) indicates *p* < 0.001. Within-group changes were evaluated using the Wilcoxon signed-rank test (CCECAI) and paired-samples *t*-tests (faecal calprotectin).

In dogs administered probiotics combined with fibre, the mean CCECAI score decreased from 6.67 ± 1.41 at baseline to 2.78 ± 3.31 after treatment (*p* = 0.016). A final CCECAI score of ≤3 was achieved in 7 of 9 dogs (77.8%). Faecal calprotectin concentrations decreased significantly from 45.36 ± 11.20 ng/mL to 17.37 ± 11.10 ng/mL (*p* = 0.000381). Serum folate concentrations increased from 7.94 ± 5.15 ng/mL to 10.08 ± 2.06 ng/mL, and serum cobalamin concentrations increased from 284.67 ± 198.87 pg/mL to 453.00 ± 163.90 pg/mL; neither change was statistically significant. A comparison of the baseline and post-treatment values of CCECAI and calprotectin in dogs administered metronidazole or probiotics with fibre is given in Figure 3.

The proportion of dogs that achieved a final CCECAI score of ≤3 did not differ significantly between the metronidazole and probiotics-plus-fibre groups (87.5% vs. 77.8%, respectively; Fisher’s exact test, *p* = 1.00). Baseline-adjusted between-treatment analyses showed no statistically significant differences in CCECAI, faecal calprotectin, serum folate, or serum cobalamin. For CCECAI, the baseline-adjusted ordinal regression model showed no significant associations between treatment group and final CCECAI score (OR = 0.70; 95% CI, 0.12–3.99; *p* = 0.685). The adjusted between-group differences were 6.50 ng/mL for faecal calprotectin (95% CI, −7.87 to 20.87; *p* = 0.375), −1.40 ng/mL for serum folate (95% CI, −4.21 to 1.40; *p* = 0.327), and −12.24 pg/mL for serum cobalamin (95% CI, −141.04 to 116.55; *p* = 0.852). Overall, no statistically significant difference in treatment response was demonstrated between metronidazole and probiotics combined with fibre for any of the evaluated outcomes. Detailed baseline and post-treatment values, within-group changes, effect estimates, and 95% confidence intervals are presented in Table 4.

### 3.3. Analysis of Changes in CCECAI Scores and Comparison of Selected Parameters Following the Use of Budesonide-Containing and Prednisone-Containing Treatments

In this part of the study, we evaluated the effects of two different treatment protocols—the prednisone-containing treatment (*n* = 4) and budesonide-containing treatment (*n* = 5) on changes in the CCECAI clinical index, folate and cobalamin in the monitored patients. No statistically significant baseline differences were detected between the prednisone and budesonide groups in age, sex distribution, CCECAI score, faecal calprotectin, serum folate, or serum cobalamin concentrations (all *p* > 0.05). The baseline characteristics of the treatment groups are given in Table 5.

In dogs administered the prednisone-containing treatment, the mean CCECAI score decreased from 12.00 ± 3.16 at baseline to 2.25 ± 1.50 after treatment, corresponding to a mean change of −9.75 points (95% CI, −13.03 to −6.47). Among the four budesonide-containing treatment dogs with complete baseline and post-treatment data, the mean CCECAI score decreased from 13.75 ± 3.20 to 1.50 ± 1.73, corresponding to a mean change of −12.25 points (95% CI, −19.05 to −5.45).

Faecal calprotectin decreased from 49.46 ± 4.25 to 20.99 ± 12.23 ng/mL in the prednisone-containing-treatment group and from 49.47 ± 5.36 to 14.73 ± 7.32 ng/mL in the four evaluable dogs administered the budesonide-containing treatment.

Serum folate increased from 5.00 ± 2.20 to 11.33 ± 1.65 ng/mL following prednisone-containing treatment and from 5.30 ± 2.43 to 10.10 ± 1.66 ng/mL following budesonide-containing treatment. Serum cobalamin increased from 194.25 ± 38.59 to 414.00 ± 31.38 pg/mL in the prednisone-containing-treatment group and from 186.75 ± 27.18 to 442.25 ± 31.83 pg/mL in the budesonide-containing-treatment group.

**Figure 4:**
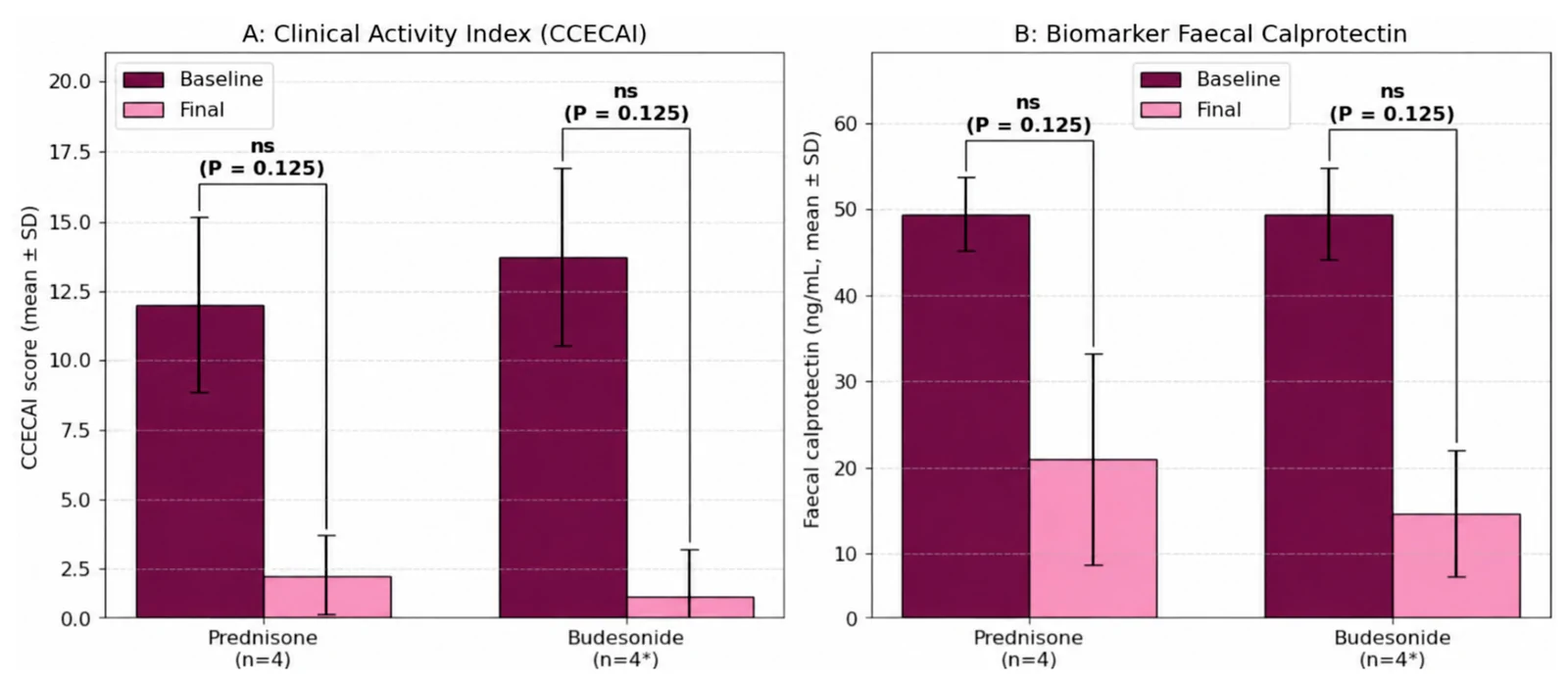
Clinical and biomarker responses to prednisone-containing and budesonide-containing treatments. Comparison of baseline (dark purple) and post-treatment (light pink) values in severe cases managed with prednisone-containing (*n* = 4) or budesonide-containing (*n* = 4) treatments. (**A**) Canine Chronic Enteropathy Clinical Activity Index (CCECAI) scores. (**B**) Faecal calprotectin concentrations (ng/mL). Data are presented as means ± standard deviations (SDs). Due to the very small subgroup sizes, numerical reductions did not reach statistical significance (ns, *p* = 0.125) when evaluated by the exact Wilcoxon signed-rank test. * Note: One dog from the initial budesonide-containing-treatment group (*n* = 5) died before the final assessment and was excluded from the paired analysis.

A comparison of the baseline and post-treatment values of CCECAI and calprotectin in dogs administered the prednisone-containing or budesonide-containing treatment is given in Figure 4.

Five dogs received the budesonide-containing treatment. Four dogs completed the planned follow-up and achieved a final CCECAI score of ≤3, whereas one dog failed to respond clinically and died before the final assessment. Accordingly, clinical remission was achieved in 4 of 5 dogs (80%) in the budesonide-containing-treatment group. Post-treatment CCECAI and biomarker concentrations were available for four dogs and were therefore used for analyses requiring paired baseline and post-treatment measurements. In the prednisone-containing-treatment group, all four dogs completed the follow-up and achieved a final CCECAI score of ≤3 (4/4, 100%). The difference in the remission rate between the groups was not statistically significant (Fisher’s exact test, *p* = 1.00).

Owing to the very small corticosteroid subgroup sizes (*n* = 4 per treatment), the findings were interpreted primarily descriptively and are considered exploratory. Both the prednisone-containing- and budesonide-containing-treatment groups showed numerical improvements in CCECAI and several laboratory variables during the treatment period. Numerically greater reductions in CCECAI and faecal calprotectin were observed in the budesonide-containing-treatment group; however, the small sample size precludes reliable conclusions regarding differences in efficacy between the two corticosteroid-containing treatments. Detailed baseline and post-treatment values, within-group changes, effect estimates, and 95% confidence intervals are presented in Table 6.

## 4. Discussion

Monitoring of 50 patients over a four-week period showed that both the hydrolysed diet and the novel-protein diet resulted in a significant improvement in the clinical conditions of dogs with chronic enteropathy. Both diets led to a statistically significant reduction in the post-treatment CCECAI score. Among dogs fed the novel-protein diet, a clinical remission rate (final CCECAI score ≤ 3) of 64% (16/25) was recorded, whereas the corresponding rate among dogs fed the hydrolysed diet was 60% (15/25). These high success rates fully confirm the key role of elimination diets as first-line therapy. Our results are consistent with data from other studies. According to [42], strict dietary therapy leads to resolution of clinical signs in 60–88% of dogs with chronic enteropathy, with the response usually occurring within the first 14–30 days, corresponding to the period evaluated in our study.

A direct comparison of the two diets showed that the novel-protein diet produced slightly better numerical results for several parameters. The mean reduction in the CCECAI score was marginally greater with the novel-protein diet than with the hydrolysed diet, at 3.40 and 3.24 points, respectively. Despite this numerical difference, our baseline-adjusted statistical analysis using an ordinal logistic regression model did not demonstrate a significant difference between the two groups (OR = 0.74; 95% CI, 0.27–2.05; *p* = 0.562). According to [3], which compared long-term outcomes in 203 dogs with chronic enteropathy, no statistically significant difference in clinical response was found between dogs fed a hydrolysed diet and those fed an elimination diet based on a novel protein. Our findings indicate that both dietary approaches were associated with clinical improvement, and that the choice between them in clinical practice may be adapted to the individual preferences, tolerance, and specific medical history of each patient.

An interesting finding in our study population was that the condition of one dog fed the hydrolysed diet deteriorated. Although hydrolysed diets are designed to minimise the immune response by breaking proteins down into smaller peptides, this process is not always completely effective. According to [43], the process of enzymatic hydrolysis cannot guarantee the complete removal of all allergenic epitopes in certain commercial diets. In practice, this means that when a dog has a marked hypersensitivity to chicken protein and is administered a chicken-based hydrolysed diet, a mild allergic or inflammatory reaction may still occur.

We also compared the efficacy of two different treatment protocols: the administration of antibiotics and the administration of a combination of probiotics and fibre. In both groups, a marked and statistically significant reduction in the CCECAI score was observed, from a baseline of 6.38 ± 1.30 to a final score of 2.63 ± 3.54 in the metronidazole group (*p* = 0.031) and from 6.67 ± 1.41 to 2.78 ± 3.31 in the probiotics and fibre group (*p* = 0.016). This reduction demonstrates the efficacy of both approaches.

The absence of a statistically significant difference in the baseline-adjusted final score between antibiotics and probiotics combined with fibre is clinically important and fully consistent with the latest findings. According to [44], which compared probiotic administration with a combination of metronidazole and prednisolone in dogs with chronic enteropathy, both groups show almost identical clinical improvements and reductions in the disease activity index. Our results show substantial clinical improvement with both approaches. These findings support further investigation of probiotics combined with fibre as a potential non-antibiotic therapeutic strategy.

A more detailed evaluation of individual responses showed that the antibiotic-treated group contained a slightly higher proportion of dogs that achieved clinical remission (final CCECAI ≤ 3), at 87.5% (7/8), which can be compared with 77.8% (7/9) in the probiotics group, though this difference was not statistically significant. Metronidazole may induce short-term clinical improvement in some dogs with chronic enteropathy; however, its broad effects on the intestinal microbiota may also contribute to dysbiosis.

However, routine empirical antibiotic administration for nonspecific chronic diarrhoea is increasingly discouraged in veterinary gastroenterology. Studies [45,46] have demonstrated that antimicrobial treatment can induce substantial and prolonged intestinal dysbiosis, reduce the diversity of beneficial bacterial populations, and contribute to the development of antimicrobial resistance. In addition, clinical relapse after antimicrobial withdrawal is common. One study [3] reported that all 33 dogs that initially responded to metronidazole subsequently relapsed within 6–12 months. Similar or even more rapid recurrence has been described after tylosin treatment; [47] reported that 86% of dogs relapsed within 30 days after discontinuation, and 88% relapsed within the first two months.

These findings have important implications for antimicrobial stewardship. The recent ACVIM-endorsed consensus [39] does not recommend empirical antimicrobial treatment in dogs suspected of having CIE and emphasises that antibiotics should be reserved for selected cases in which other therapeutic approaches have failed. Microbiome-directed strategies that do not rely on antimicrobials may therefore be particularly relevant within a step-up therapeutic approach. In the present study, no comparable pattern of early relapse was observed during the available follow-up in dogs administered probiotics combined with fibre; however, long-term treatment stability was not formally compared between groups, and the small subgroup sizes preclude conclusions regarding comparative efficacy.

Probiotics combined with fibre may represent a potential antimicrobial-sparing strategy for selected dogs with CIE. Fermentable fibre can act as a substrate for intestinal microorganisms and promote the production of short-chain fatty acids, including butyrate, which contribute to enterocyte energy metabolism and intestinal barrier homeostasis. Nevertheless, the clinical efficacy of probiotic and prebiotic interventions in CIE is variable and may depend on the specific product, microbial strains, fibre source, and patient population. Thus, our findings should be interpreted as supportive of further investigation of probiotic-plus-fibre treatment. Reducing unnecessary antimicrobial exposure while prioritising dietary and non-antibiotic microbiome-directed interventions is consistent with contemporary antimicrobial stewardship principles and current recommendations for the management of canine CIE [39].

When evaluating the therapeutic response to corticosteroid-containing treatments, profound clinical improvements were observed in both groups. In dogs administered the prednisone-containing treatment (*n* = 4), the mean CCECAI score decreased from 12.00 ± 3.16 to 2.25 ± 1.50 (mean change of −9.75 points), and complete clinical remission (score ≤ 3) was achieved in 100% (4/4) of patients. For the dogs that completed the follow-up in the budesonide-containing treatment group (*n* = 4), the mean CCECAI score decreased from 13.75 ± 3.20 to 1.50 ± 1.73 (a mean change of −12.25 points), with a remission rate of 80% (4/5) when the dog that probably failed to respond and died is included. Due to the very small subgroup sizes, within-group reductions did not reach statistical significance, and direct comparison of changes between groups similarly revealed no significant differences.

Our finding that both treatments provided the capacity to suppress clinical signs is consistent with the largest randomised clinical study to date that directly compared these treatments. According to [15], both medications resulted in significant and mutually comparable reductions in CIBDAI scores. The authors found no statistically significant difference in the remission rate, which was 78% for budesonide and 69% for prednisone. The same conclusion was reached in a more recent review. According to one study [48], the currently available evidence-based medical data do not support the conclusion that budesonide is unequivocally clinically superior to conventional prednisone for the suppression of CIE symptoms in dogs. Nevertheless, from pharmacological and pathophysiological perspectives, the response to budesonide-containing treatment observed in our study is clinically plausible.

Budesonide is a locally acting glucocorticoid with an extremely high affinity for glucocorticoid receptors. It has been reported to be up to 15 times more potent than prednisolone. According to [36], following oral administration in dogs, the medication is rapidly absorbed and accumulates directly within the cells of the intestinal mucosa, thereby providing a strong and direct anti-inflammatory effect at the site of the disease.

The slightly lower clinical score average in the budesonide-containing-treatment group might be masked by sample limitations, but systemic adverse effects must be factored in for conventional prednisone. According to [49], marked polyuria, polydipsia, excessive panting, and muscle weakness occur frequently during prednisone treatment.

Our findings regarding the efficacy of budesonide-containing treatment reflect the latest therapeutic trends. According to [50], budesonide is currently used successfully for the long-term control of symptoms in dogs with chronic enteropathy. After the initial control of inflammation, the dose can be safely reduced by up to 75% over the long term in most dogs. Our findings indicate that were substantial numerical clinical improvements in the dogs that completed the full course of budesonide-containing treatment. However, because of the very small sample size and the absence of a statistically significant difference compared with the prednisone-containing treatment, these findings are considered exploratory and do not establish the superiority of the budesonide-containing treatment. Practical considerations should also be considered, as the cost and availability of budesonide may limit its use compared with prednisone, especially when prolonged treatment is required. Therefore, the choice between budesonide and prednisone should involve not only clinical efficacy but also potential systemic effects, treatment duration, availability, and financial considerations for the owner.

Comparison of baseline and post-treatment faecal calprotectin concentrations showed that both the hydrolysed diet and the novel-protein diet resulted in a statistically significant reduction in this inflammatory biomarker. The fact that both diets effectively reduced the degree of local intestinal inflammation further confirms their key role in the treatment of chronic enteropathies. A direct post-treatment comparison between the two diets revealed no statistically significant differences in the baseline-adjusted linear model. According to [51], both diets evaluated in their study—a hydrolysed diet and a highly digestible control diet—produced comparable and successful initial clinical improvements and resolution of inflammation. The successful reduction in calprotectin achieved with both dietary approaches in our study supports the findings reported in [26], which stated that faecal calprotectin concentrations decrease significantly following the successful introduction of appropriate treatment and therefore serve as a reliable indicator of the resolution of inflammation, irrespective of the type of effective diet selected for the patient.

In dogs fed the novel-protein diet, folate increased slightly from 11.20 ± 4.06 to 12.46 ± 4.53 ng/mL, whereas it decreased from 12.19 ± 6.52 to 9.39 ± 3.62 ng/mL in the hydrolysed diet group. Although the baseline-adjusted analysis yielded an unadjusted *p* value of 0.014 for the between-group difference (+3.09 ng/mL; 95% CI, 0.62–5.56), this difference did not remain statistically significant after correction for multiple comparisons (Holm-adjusted *p* = 0.056). According to [51], who evaluated the effects of hydrolysed diets in patients with chronic enteropathy, the introduction of treatment also resulted in a reduction in serum folate concentrations, which subsequently normalised only after targeted supplementation. The mechanism underlying this finding remains unclear. The author suggested that changes in folate requirements during recovery from chronic enteropathy may reflect alterations in folate production and utilisation by the intestinal microbiome, host metabolism, and catabolism. Therefore, although diet-induced changes in the intestinal microbiome may represent one possible explanation for the divergent folate trends observed in our study, this mechanism was not directly investigated and is considered hypothetical.

When evaluating serum cobalamin, we found that the post-treatment changes associated with both diets were minimal and statistically non-significant. The mean concentration changed from 483.36 ± 255.01 to 438.24 ± 143.54 pg/mL with the novel-protein diet and from 400.28 ± 199.96 to 445.60 ± 145.33 pg/mL with the hydrolysed diet. Comparison of the baseline-adjusted treatment effects revealed no statistically significant differences between the two therapies. From a clinical perspective, however, it is important to note that dietary modification alone is generally insufficient to produce a rapid increase in cobalamin concentrations when a deficiency is already present.

A previous study [23] demonstrated an association between lower serum cobalamin concentrations and more severe endoscopic and histological ileal lesions in dogs with chronic inflammatory enteropathy. Nevertheless, stable or normal serum cobalamin concentrations cannot be considered evidence of absent or limited ileal involvement. Therefore, in the present study, the cobalamin findings are interpreted as suggesting the absence of marked impairment of cobalamin absorption rather than as a direct indicator of the extent of ileal disease.

According to [52], active oral or parenteral supplementation of this vitamin is generally required to achieve a significant increase and replenish tissue stores in patients with chronic enteropathy.

Comparison of baseline and post-treatment faecal calprotectin concentrations in the antibiotic and probiotic groups showed that both treatment protocols resulted in a statistically significant reduction in this biomarker, dropping by −30.76 ng/mL under metronidazole and −28.00 ng/mL under probiotics and fibre. A baseline-adjusted comparison of the two therapies revealed no statistically significant differences between them. This finding suggests that non-pharmacological modulation of the microbiome may have the potential to reduce faecal calprotectin. Our observations are consistent with the latest findings in veterinary gastroenterology. According to one study [53], the administration of synbiotics in dogs with chronic enteropathy resulted in a significant reduction in faecal calprotectin after only two weeks. The authors attributed this effect to restoration of a healthy mucosal barrier and a reduction in the local inflammatory infiltrate.

The analysis of changes in serum folate concentrations revealed several interesting differences. In dogs treated with antibiotics, a statistically significant increase in the mean post-treatment folate concentration was observed, rising from 7.41 ± 2.53 to 11.45 ± 2.99 ng/mL (*p* = 0.011). In contrast, the increase in the probiotics-and-fibre group (from 7.94 ± 5.15 to 10.08 ± 2.06 ng/mL) was mild and statistically non-significant. However, the baseline-adjusted difference between the two treatments was not significant (−1.40 ng/mL; *p* = 0.327). This trend has also been confirmed by other authors. According to [54], the successful initiation of treatment in dogs with chronic enteropathy resulted in a significant increase in serum folate concentrations, which directly correlated with the resolution of clinical signs. The more significant within-group increase in folate observed in our antibiotic-treated group may, however, also have been associated with the effect of antibiotics on the intestinal microbiota. According to [55], antibiotics cause dysbiosis and alter the composition of individual bacterial populations. Some dysbiotic bacteria may temporarily predominate in the intestine following administration of particular antibiotics and can directly synthesise folate.

When evaluating serum cobalamin concentrations, we found a slight numerical decrease in metronidazole (−32.88 pg/mL; *p* = 0.795), while the probiotics and fibre group demonstrated a substantial numerical increase from 284.67 ± 198.87 to 453.00 ± 163.90 pg/mL (+168.33 pg/mL; *p* = 0.137). The baseline-adjusted between-group difference did not reach statistical significance. The negative effects of antibiotics on nutrient absorption and microbial balance in the distal intestine are also indirectly supported by other extensive studies. According to [56,57], the administration of antibiotics such as metronidazole in dogs causes marked changes in the faecal microbiome, disrupting important metabolic pathways and potentially negatively affecting overall intestinal absorption.

Monitoring of faecal calprotectin in the corticosteroid-containing subgroups demonstrated substantial numerical reductions in both arms, decreasing by −28.47 ng/mL in the prednisone-containing-treatment group and −34.74 ng/mL in the budesonide-containing-treatment group. Direct comparison of individual changes between the treatment groups showed no statistically significant differences due to the limited power of the small cohorts. Nevertheless, both treatments showed a capacity to suppress local inflammation. According to [37], budesonide is characterised by a high affinity for local glucocorticoid receptors and undergoes first-pass hepatic metabolism following absorption, exerting a strong localised effect on the intestinal mucosal surface at the site of inflammation while minimising extensive systemic availability.

A similar stabilising trend was observed when serum folate concentrations were evaluated. In both corticosteroid-containing groups, folate concentrations increased towards the physiological range (+6.33 ng/mL for the prednisone-containing-treatment group, +4.80 ng/mL for the budesonide-containing-treatment group; both within-group *p* = 0.125), with no significant difference between changes. According to [22], hypofolataemia in chronic enteropathies is a direct consequence of malabsorption in the proximal part of the intestine.

An important clinical finding was obtained from the evaluation of the final serum cobalamin concentration. Both treatments were successful numerically, with the prednisone-containing treatment increasing cobalamin by +219.75 pg/mL and the budesonide-containing treatment increasing it by +255.50 pg/mL (both *p* = 0.125; between-group *p* = 0.686). According to [19,58], chronic inflammatory enteropathies in dogs cause significant changes and damage to these ileal transporters, representing the primary cause of cobalamin malabsorption.

From a practical clinical perspective, the duration and monitoring of dietary trials are important components of the sequential management of CIE. The recently published ACVIM-endorsed consensus [39] recommends exclusive provision of an appropriately selected therapeutic diet for at least 2 weeks to assess the initial response, with clinical activity monitored at least weekly using standardised indices such as the CCECAI or CIBDAI. Longitudinal assessment should additionally include body weight, body and muscle condition scores, faecal consistency, quality of life, and relevant laboratory abnormalities. If clinically feasible, more than one adequately performed dietary trial should be considered before treatment escalation. Furthermore, when a therapeutic diet induces clinical remission, the consensus recommends maintaining that diet for at least 12 weeks before attempting a transition back to the previous or another non-therapeutic diet, as a relapse may occur after a dietary rechallenge. In the present study, dietary response was assessed after a 4-week treatment period using the CCECAI together with faecal calprotectin, serum folate, and serum cobalamin concentrations. This follow-up period was therefore sufficient to evaluate the initial therapeutic response but does not permit us to draw conclusions regarding the durability of remission or the risk of relapse after discontinuation of the therapeutic diet. Nevertheless, the fundamental therapeutic sequence used in the present study—with dietary modification as the first step, followed by microbiome-directed intervention and, when required, corticosteroid therapy—is broadly consistent with the stepwise management strategy subsequently formalised by the consensus. Thus, although differences in monitoring frequency, the number of consecutive dietary trials, and the duration of long-term follow-up remain, the present protocol reflects several principles that have since been incorporated into contemporary recommendations for CIE management.

## 5. Conclusions

This study provides real-world clinical data on the sequential management of canine chronic inflammatory enteropathy using a step-up therapeutic approach implemented in routine veterinary practice. Dietary intervention represented the first therapeutic step and was associated with significant clinical improvement and reductions in faecal calprotectin with both novel-protein and hydrolysed diets. In dogs requiring further treatment, both metronidazole and probiotics combined with fibre were associated with marked clinical and biomarker improvement. These findings support the increasing clinical relevance of non-antibiotic microbiome-directed strategies, particularly in the context of antimicrobial stewardship. In dogs requiring corticosteroid therapy, both prednisone-containing and budesonide-containing treatments were associated with substantial numerical clinical improvement; however, the very small subgroup sizes preclude conclusions regarding the superiority of either corticosteroid.

An important strength of the present study is that it reflects treatment decision-making under real clinical conditions, where therapeutic interventions must often be introduced sequentially and adapted according to individual patient responses. Although this pragmatic design inevitably introduces limitations, it also reflects the challenges encountered in everyday management of canine chronic inflammatory enteropathy. Importantly, the sequential strategy applied in this cohort is broadly consistent with current recommendations that prioritise dietary management, limit unnecessary antimicrobial exposure, and reserve immunomodulatory therapy for dogs with insufficient responses to earlier therapeutic steps. Therefore, rather than establishing definitive comparative superiority of individual treatments, the present findings provide complementary real-world evidence supporting a structured, individualised, and progressively escalated therapeutic approach to canine chronic inflammatory enteropathy and highlight the need for larger prospective controlled studies to confirm the observed treatment-associated effects. Future research should particularly include adequately powered randomised trials directly comparing budesonide and prednisone as well as long-term studies evaluating microbiome-directed, antimicrobial-sparing strategies, including probiotics and fibre, with emphasis on sustained remission, relapse rates, and long-term disease control.

### Limitations

- Only metronidazole was evaluated as an antimicrobial therapy in the present study.
- The sample size was small, and there was an unequal distribution of dogs among the different therapeutic response groups.
- Endoscopy and histopathological examination were performed only in dogs with very severe chronic inflammatory enteropathy.
- In dogs with very severe chronic inflammatory enteropathy, corticosteroid therapy was administered concurrently with previously initiated dietary, probiotic, and fibre interventions.
- The allocation of dogs to treatment groups was done alternately.
- The study has a relatively short follow-up period.

## Figures and Tables

**Table 1:** Baseline characteristics of treatment diet groups.

Baseline Characteristic	Novel-Protein Diet (*n* = 25)	Hydrolysed Diet (*n* = 25)	*p* Value
Age (years)	5.16 ± 3.18	6.84 ± 4.17	0.148
Sex (F/M)	15/10	11/14	0.396
CCECAI score	5.96 ± 1.90	6.32 ± 1.57	0.360
Faecal calprotectin (ng/mL)	32.52 ± 11.39	35.40 ± 14.13	0.509
Serum folate (ng/mL)	11.20 ± 4.06	12.19 ± 6.52	0.523
Serum cobalamin (pg/mL)	483.36 ± 255.01	400.28 ± 199.96	0.256

Data are presented as means ± standard deviations (SDs), except for sex, which is presented as the number of females/males (F/M). Between-group comparisons were performed using the Mann–Whitney U test for variables that did not meet the assumption of normality (age, CCECAI, faecal calprotectin, and cobalamin), the independent-samples *t*-test for serum folate, and Fisher’s exact test for sex distribution.

**Table 2:** Clinical and biomarker responses to dietary treatments.

Outcome	Novel-Protein Baseline	Novel-Protein Final	Change (95% CI)	***p***
CCECAI	5.96 ± 1.90	2.56 ± 2.90	−3.40 (−4.51 to −2.29)	1.45 × 10^−6^
Faecal calprotectin (ng/mL)	32.52 ± 11.39	17.39 ± 11.86	−15.13 (−22.60 to −7.65)	0.000336
Serum folate (ng/mL)	11.20 ± 4.06	12.46 ± 4.53	+1.26 (−0.95 to 3.48)	0.251
Serum cobalamin (pg/mL)	483.36 ± 255.01	438.24 ± 143.54	−45.12 (−184.56 to 94.32)	0.511
Outcome	Hydrolysed Baseline	Hydrolysed Final	Change (95% CI)	***p***
CCECAI	6.32 ± 1.57	3.08 ± 3.26	−3.24 (−4.50 to −1.98)	1.85 × 10^−5^
Faecal calprotectin (ng/mL)	35.40 ± 14.13	17.08 ± 10.97	−18.32 (−25.02 to −11.63)	8.15 × 10^−6^
Serum folate (ng/mL)	12.19 ± 6.52	9.39 ± 3.62	−2.80 (−6.06 to 0.46)	0.089
Serum cobalamin (pg/mL)	400.28 ± 199.96	445.60 ± 145.33	+45.32 (−61.07 to 151.71)	0.388
Outcome	Baseline-Adjusted Treatment Effect	95% CI		***p***
CCECAI	OR = 0.74	0.27–2.05		0.562
Faecal calprotectin	+0.30 ng/mL	−6.36 to 6.97		0.929
Serum folate	+3.09 ng/mL	+0.62 to +5.56		0.014
Serum cobalamin	+6.01 pg/mL	−80.00 to +92.03		0.891

Data are presented as means ± standard deviations (SDs). Changes are expressed as mean differences between post-treatment and baseline values with 95% confidence intervals. Within-group changes in CCECAI were evaluated using the Wilcoxon signed-rank test because CCECAI is an ordinal clinical score. Changes in laboratory biomarkers were evaluated using paired-samples *t*-tests when the distribution of paired differences was compatible with normality. Between-treatment differences in CCECAI were assessed using a baseline-adjusted ordinal logistic regression model. Laboratory outcomes were compared using baseline-adjusted linear regression models with robust standard errors. Treatment effects are reported with 95% confidence intervals.

**Table 3:** Baseline characteristics of treatment groups administered antibiotics and probiotics with fibre.

Baseline Characteristic	Metronidazole (*n* = 8)	Probiotics + Fibre (*n* = 9)	*p* Value
Age (years)	6.38 ± 2.56	9.33 ± 4.87	0.146
Sex (F/M), *n*	0/8	4/5	0.082
CCECAI score	6.38 ± 1.30	6.67 ± 1.41	0.666
Faecal calprotectin (ng/mL)	40.86 ± 7.82	45.36 ± 11.20	0.123
Serum folate (ng/mL)	7.41 ± 2.53	7.94 ± 5.15	0.795
Serum cobalamin (pg/mL)	457.75 ± 281.48	284.67 ± 198.87	0.093

Data are presented as means ± standard deviations (SDs), except for sex, which is presented as the number of females/males (F/M). Between-group comparisons were performed using the independent-samples *t*-test for variables meeting the assumptions of normality and homogeneity of variance, the Mann–Whitney U test for variables not meeting the assumption of normality, and Fisher’s exact test for sex distribution. F—female, M—male.

**Table 4:** Clinical and biomarker responses to metronidazole and probiotics combined with fibre.

Outcome	Metronidazole Baseline	Metronidazole Final	Change (95% CI)	*p*	Probiotics + Fibre Baseline	Probiotics + Fibre Final	Change (95% CI)	*p*
CCECAI	6.38 ± 1.30	2.63 ± 3.54	−3.75 (−6.53 to −0.97)	0.031	6.67 ± 1.41	2.78 ± 3.31	−3.89 (−6.11 to −1.67)	0.016
Faecal calprotectin (ng/mL)	40.86 ± 7.82	10.10 ± 11.04	−30.76 (−41.41 to −20.11)	0.000246	45.36 ± 11.20	17.37 ± 11.10	−28.00 (−39.02 to −16.97)	0.000381
Serum folate (ng/mL)	7.41 ± 2.53	11.45 ± 2.99	+4.04 (+1.25 to +6.82)	0.011	7.94 ± 5.15	10.08 ± 2.06	+2.13 (−2.13 to +6.40)	0.164
Serum cobalamin (pg/mL)	457.75 ± 281.48	424.88 ± 109.51	−32.88 (−320.34 to +254.59)	0.795	284.67 ± 198.87	453.00 ± 163.90	+168.33 (−66.79 to +403.45)	0.137
Outcome	Adjusted effect: probiotics + fibre vs. metronidazole						95% CI	*p*
CCECAI	OR = 0.70						0.12–3.99	0.685
Faecal calprotectin	+6.50 ng/mL						−7.87 to 20.87	0.375
Serum folate	−1.40 ng/mL						−4.21 to 1.40	0.327
Serum cobalamin	−12.24 pg/mL						−141.04 to 116.55	0.852

Data are presented as means ± standard deviations (SDs). Changes are expressed as the mean difference between post-treatment and baseline values with 95% confidence intervals (CIs). Within-group changes in CCECAI were evaluated using the Wilcoxon signed-rank test because CCECAI is an ordinal clinical score. Paired-samples *t*-tests were used for laboratory variables when the distribution of paired differences was compatible with normality; otherwise, the Wilcoxon signed-rank test was applied. A negative change indicates a decrease from baseline, whereas a positive change indicates an increase.

**Table 5:** Baseline characteristics of treatment groups administered prednisone-containing and budesonide-containing treatments.

Baseline Characteristic	Prednisone-Containing (*n* = 4)	Budesonide-Containing (*n* = 5)	*p* Value
Age (years)	9.25 ± 5.56	5.60 ± 2.61	0.413
Sex (F/M), *n*	1/3	3/2	0.524
CCECAI score	12.00 ± 3.16	14.40 ± 3.13	0.190
Faecal calprotectin (ng/mL)	49.46 ± 4.25	50.94 ± 5.69	0.730
Serum folate (ng/mL)	5.00 ± 2.20	5.04 ± 2.18	1.000
Serum cobalamin (pg/mL)	194.25 ± 38.59	179.40 ± 28.71	0.730

Data are presented as means ± standard deviations (SDs), except for sex, which is presented as the number of females/males (F/M). Owing to the small subgroup sizes, baseline continuous variables were compared using the exact Mann–Whitney U test, while sex distribution was compared using Fisher’s exact test. CCECAI, Canine Chronic Enteropathy Clinical Activity Index; F, female; M, male.

**Table 6:** Clinical and biomarker responses to treatment with budesonide-containing and prednisone-containing treatments.

Outcome	Prednisone-Containing Baseline	Prednisone-Containing Final	Change (95% CI)	*p*	Budesonide-Containing Baseline	Budesonide-Containing Final	Change (95% CI)	*p*
CCECAI	12.00 ± 3.16	2.25 ± 1.50	−9.75 (−13.03 to −6.47)	0.125	13.75 ± 3.20	1.50 ± 1.73	−12.25 (−19.05 to −5.45)	0.125
Faecal calprotectin (ng/mL)	49.46 ± 4.25	20.99 ± 12.23	−28.47 (−49.45 to −7.50)	0.125	49.47 ± 5.36	14.73 ± 7.32	−34.74 (−51.01 to −18.48)	0.125
Serum folate (ng/mL)	5.00 ± 2.20	11.33 ± 1.65	+6.33 (+1.25 to +11.40)	0.125	5.30 ± 2.43	10.10 ± 1.66	+4.80 (+1.03 to +8.57)	0.125
Serum cobalamin (pg/mL)	194.25 ± 38.59	414.00 ± 31.38	+219.75 (+113.22 to +326.28)	0.125	186.75 ± 27.18	442.25 ± 31.83	+255.50 (+188.67 to +322.33)	0.125
Outcome	Prednisone-Containing Change	Budesonide-Containing Change	*p*					
CCECAI	−9.75	−12.25	0.343					
Faecal calprotectin	−28.47	−34.74	0.486					
Serum folate	+6.33	+4.80	0.686					
Serum cobalamin	+219.75	+255.50	0.686					

Data are presented as means ± standard deviations (SDs). Changes are expressed as the mean difference between post-treatment and baseline values with 95% confidence intervals (CIs). Owing to the very small subgroup sizes (*n* = 4 per group), within-group changes were evaluated using the exact Wilcoxon signed-rank test, and between-group comparisons of individual changes were performed using the exact Mann–Whitney U test. A negative change indicates a decrease from baseline, whereas a positive change indicates an increase. CCECAI, Canine Chronic Enteropathy Clinical Activity Index. Five dogs received budesonide-containing treatment; however, complete post-treatment data were available for four dogs. One dog did not respond to treatment and had no final CCECAI or biomarker measurements; therefore, this dog was included in the baseline characteristics but excluded from paired analyses requiring post-treatment measurements.

## Data Availability

Dataset available on request from the authors.
